# Novel Electrically Conductive Cellulose Nanocrystals with a Core-Shell Nanostructure Towards Biodegradable Electronics

**DOI:** 10.3390/nano13040782

**Published:** 2023-02-20

**Authors:** Hatem Abushammala, Jia Mao

**Affiliations:** 1 Environmental Health and Safety Program, College of Health Sciences, Abu Dhabi University, Abu Dhabi P.O. Box 59911, United Arab Emirates; 2 Fraunhofer Institute for Wood Research (WKI), Bienroder Weg 54E, 38108 Braunschweig, Germany; 3 Department of Mechanical Engineering, Al Ghurair University, International Academic City, Dubai P.O. Box 37374, United Arab Emirates

**Keywords:** cellulose, nanocrystals, toluene diisocyanate, electrical conductivity, e-waste, core-shell

## Abstract

Electronic waste (e-waste) is the fastest growing waste stream and its negative impact on the environment and human health is major because of the toxicity and non-biodegradability of its constituents. For their biodegradability and nontoxicity, bio-based materials have been proposed as potential material candidates in the field of electronics. Among these, cellulose nanocrystals (CNCs) have many interesting properties including biodegradability, high mechanical strength, and possibility to functionalize. In terms of electrical properties, CNCs are electrically insulated, limiting their potential in electronics. This work aims to build up a poly(o-toluidine)-like shell around the CNCs to render them conductive. For this goal, the surface of the CNCs was carbamated using 2,4-toluene diisocyanate through the para-isocyanates and the ortho-isocyanates were later hydrolyzed to amine groups using HCl-acidified dimethylsulfoxide. The resultant o-toluidine-like molecules on the CNC surface were then polymerized using ammonium persulfate to form an electrically conductive shell around each CNC. The resultant CNCs were then characterized for their chemical, morphological, and electrical properties. Fourier-transform infrared analysis of the CNCs at each stage confirmed the expected chemical changes upon carbamation, hydrolysis, and polymerization and X-ray diffraction confirmed the permanence of the native crystalline structure of the CNCs. The atomic force microscopy images showed that the obtained CNCs were on average slightly thicker than the original ones, possibly due to the growth of the poly(o-toluidine) shell around them. Finally, using the four-point method, the obtained CNCs were electrically conductive with a conductivity of 0.46 S/cm. Such novel electrically conductive CNCs should have great potential in a wide range of applications including electronics, sensing, and medicine.

## 1. Introduction

The impact of electronic waste (e-waste) on global warming is immense, especially with the high current demand on technological products such as mobiles and laptops and the non-degradable oil-based nature of their components, not forgetting the negative environmental impacts of their fabrication and recycling [1]. Out of the 50 million tons of e-waste that are generated annually worldwide, less than 10% is recycled and the rest ends up in dumping sites or burned, leading to massive greenhouse gas emissions and the release of hazardous materials into soil and water [2,3]. Due to their biodegradability, bio-based materials have been proposed to help in reducing the massive amounts of e-waste [4]. Moreover, if the electronic products are designed for human health solutions such as biochips for biosensing or as drug delivery systems, the biocompatibility of bio-based materials is a major advantage [5,6]. Despite the interesting properties of bio-based materials, the research on their inclusion in electronics is limited to their use as inactive substrates to hold the conductive components of electronics [7,8]. This is not surprising since bio-based materials are insulated in nature. Therefore, the development of technologies to render them conductive could foster the production of biodegradable electronics. Among bio-based materials, cellulose nanocrystals (CNCs) are rod-like nanoparticles with a thickness of 3–10 nm and a length of few hundreds of nanometers [9]. They have been extracted from cellulose fibers and directly from lignocelluloses using a wide range of methods [10,11,12,13,14,15,16,17]. CNCs have many interesting properties including high mechanical strength and surface area, the possibility to functionalize, and the ability to form liquid crystalline structures, making them very interesting for a wide range of applications [18,19,20,21]. However, CNCs are electrically insulated, limiting their potential in electronic applications despite their use with conductive polymers such as poly(aniline) and poly(pyrrole) to develop electrically conductive paper and composites [22,23]. In these reports, CNCs acted merely as a non-functional substrate that provides only mechanical integrity and flexibility. The final product was mostly a conductive paper of an isotropic mixture of CNCs and the conductive polymer. In other words, the CNCs themselves were not made conductive.

Poly(aniline), synthesized in 1862 by Henry Letheby, is the first known organic conductive material with a conductivity ranging between 0.1 and 100 S/cm. Poly(aniline) is the most famous conducting polymer for its high conductivity, lightweight, diverse nano-structures, good environmental stability, and its synthesis is considered simple and easy to control [24]. It is prepared by the oxidation of aniline in an acidic medium [25,26]. Aniline, the monomer, is produced industrially via a two-step process that involves the nitration of benzene followed by hydrogenation in the presence of a metal catalyst [27]. A wide range of oxidation agents were used for the polymerization of aniline to poly(aniline): persulfates, dichromates, and iron (III) chloride [24,28,29]. Poly(aniline) has an acid/base doping response, making it very interesting for vapor sensors and biosensors [30]. Acid dopants, such as hydrochloric acid (HCl), and perchloric acid (HClO_4_), and benzenesulfonic acid, have been used in situ to improve poly(aniline)’s conductivity. Iodine, ionic liquids, and iron (II,III) oxide (Fe_3_O_4_) have also been used as dopants [31,32,33]. The polymerization process is significant in determining the molecular weight, crystallinity, micro-/nano-structure, and electrical conductivity of poly(aniline) [34,35,36]. Poly(aniline) has three forms based on its oxidation state: emeraldine, leucomeraldine, and perniganiline. Perniganiline is the fully oxidized form of poly(aniline) and leucomeraldine is the fully reduced one. Emeraldine is the half-oxidized poly(aniline) form. When emeraldine is doped with an acid, it is called emeraldine salt, which is the only conductive form of poly(aniline) [37]. Poly(aniline) is a polychromic material; perniganiline is purple, leucomeraldine is yellow, emeraldine is blue, and the conductive emeraldine salt is green. Poly(aniline) has shown great potential for electronic components such as electrodes, supercapacitors, electrostatics, and electrochromics [38,39], and has been explored with a wide range of materials (inorganics, organics, metals, clays, surfactants, ionic liquids) to be used for photo-induced conductors [40], static dissipation [41], and dielectric materials [42]. Poly(aniline) has several derivatives that mostly vary in the number, position (ortho, meta, para), and nature of the substituents on the benzene ring such as poly(toluidine) and poly(dimethoxyaniline) [43,44] or have a certain chemical group on the amine group, which are known as N-substituted derivatives such as poly(N-methylaniline) [45,46].

**Figure 1:**
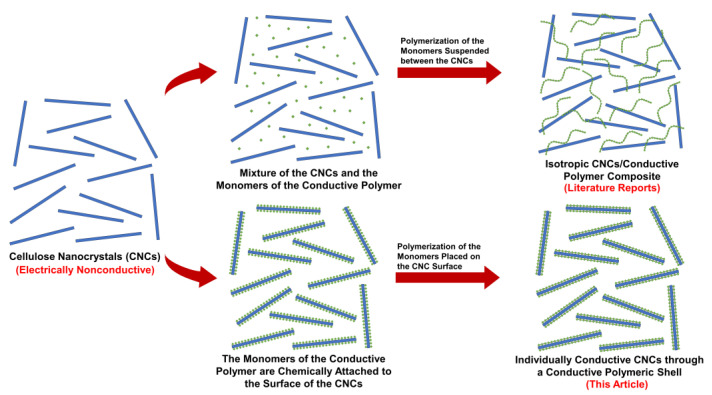
The uniqueness of the electrically conductive CNCs prepared in this article compared to those in the literature [22,23].

Inspired by the structure of poly(aniline) and its derivatives, this paper aims at engineering the surface of CNCs by growing a shell of poly(o-toluidine) around each CNC, rendering them conductive. As a result, each CNC will act as an individually conductive rod by itself, i.e., a nanowire. The process will involve attaching o-toluidine-like molecules to the CNC surface followed by polymerizing them together, forming the conductive shell (Figure 1). Such novel CNCs could have a great potential in a wide range of applications, including sensing and electronics, and could support efforts to improve the biodegradability and toxicity of electronics.

## 2. Materials and Methods

### 2.1. Materials

The CNC suspension used in this paper had a solid content of 10.4% (*w*/*w*) and was purchased from the University of Maine (Maine, USA), which was prepared using the sulfuric acid method. Acetone (>99%), chloroform (>99%), toluene (>99%), dimethylsulfoxide (DMSO) (>99.9%), triethylamine (TEA) (>99%), ammonium persulfate (APS) (>98%), and hydrochloric acid (37%) were all purchased from VWR (Darmstadt, Germany) whereas 2,4-toluene diisocyanate (2,4-TDI) (>98%) was purchased from TCI Chemicals (Eschborn, Germany) and was stored in the fridge in sealed bottles. DMSO, TEA, and toluene were stored over an A4 molecular sieve, and acetone was stored over an A3 molecular sieve. Both molecular sieves were purchased from Carl Roth (Karlsruhe, Germany) and regenerated before use.

### 2.2. Carbamation of Cellulose Nanocrystals Using 2,4-Toluene Diisocyanate

The CNCs were carbamated following the method of Habibi after minor modifications [47]. 9.6 g of 10.4% CNC suspension (equivalent to 1.0 g of dried CNCs (6.2 mmol of anhydroglucose units)) was solvent-exchanged from water to anhydrous toluene using a washing/precipitation procedure starting with anhydrous acetone (three times) then anhydrous toluene (three times). The precipitation was performed using a Sigma 3-16P centrifuge (g-force of 4472, 5000 rpm for 30 min at 25 °C) (Sigma Laborzentrifugen, Germany). After the final washing with toluene, the precipitated CNCs were transferred to a 100 mL round-bottom flask using 46 mL of anhydrous toluene. 3.3 g of 2,4-TDI (equivalent to 18.6 mmol) and 3 mL of TEA as catalyst were transferred to the reaction flask. The TDI/CNCs molar ratio was 3 and the final reaction volume was ca. 52 mL. The reaction proceeded at 35 °C for 24 h in a moisture-free environment (under nitrogen). The reaction conditions and amounts of reactants were optimized by the authors and published somewhere else [48]. The reaction mixture was then centrifuged to separate the carbamated CNCs from the unreacted TDI and TEA. The carbamated CNCs were then washed once with anhydrous toluene and twice with anhydrous DMSO to remove any residual TDI and TEA before transferred to the acidified DMSO for isocyanate hydrolysis and quantification of free isocyanates. For characterization, the carbamated CNCs were not washed with DMSO but only with toluene (three times) then oven dried overnight at 50 °C under vacuum. To assure reproducibility, the reaction was performed in duplicate. CNCs-TDI is used to refer to the carbamated CNCs.

### 2.3. Hydrolysis and Quantification of Free Isocyanates on the CNC Surface (DS_NCO_)

The free o-NCO of the grafted TDI were hydrolyzed following the method of Abushammala [49]. 100 mL of DMSO were transferred to a 200 mL beaker before being acidified to a pH of 2.47 ± 0.01 using 2 mL of 2 M HCl. The carbamated CNCs, after washed with DMSO, were transferred to the beaker. The pH started to increase because of isocyanate hydrolysis to amine groups. The hydrolysis was complete when no further increase in pH was observed (a maximum of 1 h). The degree of substitution of isocyanates (DS_NCO_) was calculated based on the final pH value upon hydrolysis using the below equation, which was developed by the authors and published somewhere else [49]. All pH measurements were recorded at a temperature of 25 ± 0.5 °C using a SevenEasy S20-KS pH meter equipped with InLab^®^ Routine Pro electrode (Mettler Toledo, Giessen, Germany). CNCs-TDN is used to refer to the carbamated CNCs after hydrolysis.

$$DS_{NCO}=\frac{mmol\;NCO}{mmol\;CNC\;Hydroxyls}=\frac{-24.3pH^{4}+290.3pH^{3}-1302.0pH^{2}+2598.7pH-1946.0}{3\;*\;\left(mass\;CNCs/162\right)\;}*100$$

### 2.4. Polymerization of the o-Toluidine Grafted on the CNC Surface

A certain amount of APS was added to the hydrolysis medium containing the CNCs after hydrolysis to start the oxidative polymerization of the generated toluidine molecules on the CNC surface. The mixture was stirred using 300 rpm at 25 °C for only one minute. At that stage, a yellow-green color could be observed. The polymerization continued without stirring for 48 h. The resultant green CNCs were collected by vacuum filtration and washed with ethanol. The product was left to dry under vacuum overnight. E-CNCs is used to refer to the final CNCs after carbamation, hydrolysis, and polymerization.

### 2.5. Determination of the Degree of Substitution of TDI (DS_TDI_)

Elemental analysis of solid samples of the CNCs at each stage of the process was conducted using a ZEISS GeminiSEM Crossbeam 340 scanning electron microscope (ZEISS, Oberkochen, Germany), equipped with an X-MaxN EDX detector (Oxford Instruments, Abingdon, UK) operating at a voltage of 10 kV. The samples in the powder form were pressed in a mold to obtain discs (diameter: 1 cm) of smooth surfaces. DS_TDI_ was determined based on the increase in the nitrogen/carbon molar ratio (R) and the equation below, which was developed by the authors and published elsewhere [49]. To assure reproducibility, the measurement was performed in duplicate.

$$DS_{TDI}=\frac{mmol\;TDI}{mmol\;CNC\;Hydroxyls}*100\%=168,796.6\;R^{4}-27,737.8\;R^{3}+2270.4\;R^{2}+36.7\;R+0.1$$

### 2.6. Structural Characterization Using Fourier Transform Infrared (FT-IR)

Samples of the CNCs at all stages of the process were characterized using Tensor 27 FT-IR spectrometer (Bruker, Billerica, MA, USA) in the 750–4000 cm^−1^ range (resolution of 2 cm^−1^) using the ATR transmission mode. The spectra were obtained as the average of 64 scans and processed using OPUS software (Version 6.5).

### 2.7. Morphological Characterization Using X-ray Diffraction (XRD)

The CNCs at all stages of the process were analyzed using a Bruker AXS D8 X-ray diffractometer (Bruker, Billerica, Massachusetts, USA) using a CuKα1 radiation, which operated at 40 kV and 40 mA. The intensities were collected at 2θ from 5 to 40° using the software Diffrac Plus XRD Commander (Version 2.6.1). Peak deconvolution was performed using the software PeakFit (version 4.12) to determine the areas of the characteristic peaks of the crystalline diffractions at 2θ of 14.9°, 16.3°, 20.4°, 22.5°, and 34.5° and the amorphous diffraction at 2θ of 18°. The crystallinity (Cr) was then calculated [50] using the below equation. To assure reproducibility, the measurement was performed in duplicate.

$$Cr=\frac{Total\;Area\;of\;Crystalline\;Diffraction\;Peaks}{Total\;Area\;of\;Crystalline\;and\;Amorphous\;Diffraction\;Peaks}*100\%$$

### 2.8. Morphological Characterization Using Atomic Force Microscopy

A drop of a diluted suspension (10^−4^%) of the CNCs at all stages of the process was deposited on a fresh mica surface then kept to air-dry overnight. The surface was imaged in the tapping mode using the atomic force microscope Agilent 5500 (Keysight Technologies, Santa Rosa, CA, USA). The silicon tips PPP-NCH (Wetzler, Nanoandmore, Germany) were used, which had a resonance frequency of ca. 350 kHz and a spring constant of ca. 50 N.m^−1^. The thickness of the CNCs was determined based on a sample size of 100 particles using the Gwyddion software (version 2.26).

### 2.9. Electrical Conductivity Measurements

**Figure 2:**
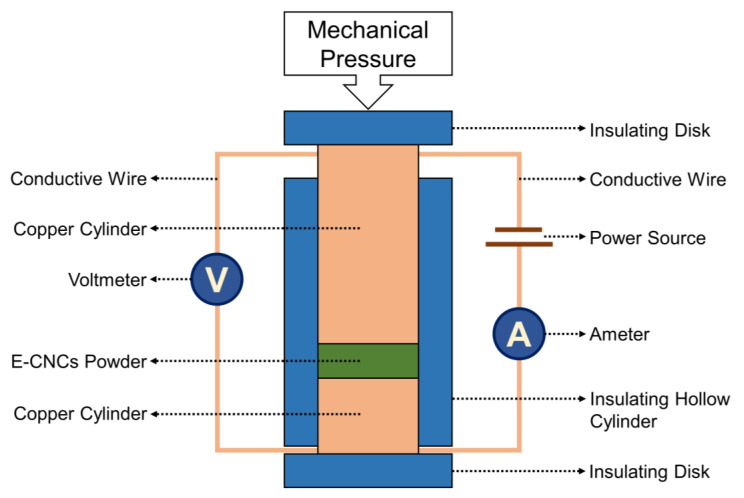
The setup used to measure the electrical conductivity of the E-CNCs powder, which is based on the four-point method.

100 mg of the conductive CNCs in powder form were placed between two copper cylinders of the same cross-sections as shown in Figure 2. The powder was subjected to 1 ton of pressure and the conductivity of the CNCs was determined following the four-point method using two multimeters (UT33C+ Digital Multimeter, UNI-T, Dongguan, China). The following formula was used to calculate the electrical conductivity:

$$\kappa =\frac{I*L}{V*A}$$

where *κ* is the electrical conductivity (S/cm), L is the thickness of the samples (cm), and A is the cross-sectional area of the samples (cm^2^). V and I represent the voltage and current measured using the two multimeters following the four-point method. The thickness of the CNC samples during the measurement was determined as the increase in the height of the setup (around 0.5 mm) using a caliber and the cross-sectional area of the samples was equivalent to the cross-sectional area of the copper cylinders (diameter of 1.5 cm). All samples were measured five times to assure reproducibility. All measurements were conducted at 25 °C and 55% relative humidity.

## 3. Results and Discussion

**Figure 3:**
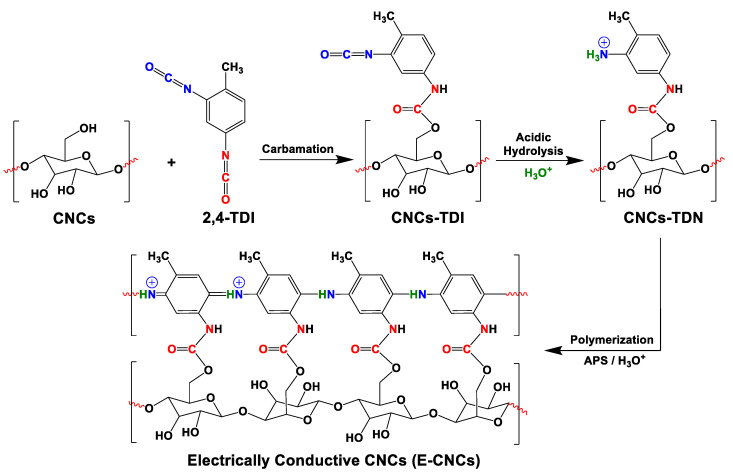
The growth of a poly(o-toluidine) nanoshell on the CNC surface using 2,4-TDI as the main reagent.

In several reports in the literature, cellulose and nanocellulose have been mixed with conductive polymers such as poly(aniline) to render them conductive. The products in these reports were mostly isotropic conductive composites, in which the cellulosic material acted merely as a non-functional substrate [23]. In this work, we aim to prepare novel electrically conductive cellulose nanocrystals (CNCs) by building up a poly(o-toluidine)-like shell on the CNC surface, making each CNC act as an individually-conductive nanowire. Such an engineered surface is expected to increase the potential of CNCs in major technological applications such as electronics and sensing. The development of the conductive shell around the CNCs started by the carbamation reaction between 2,4-TDI and the surface hydroxyls of the CNCs followed the method of Habibi, which was optimized and published by the authors elsewhere [47,48] (Figure 3). 2,4-TDI was used because of the known 5–10 times higher reactivity of its para isocyanate group compared to the ortho one [51,52,53]. Due to this difference in reactivity, the para isocyanate groups of 2,4-TDI were expected to react with the surface hydroxyl groups of CNCs whereas the ortho groups were to stay unreacted. This reaction was thoroughly optimized by the authors and published elsewhere [48]. The second step was the hydrolysis of the unreacted ortho isocyanates on the carbamated CNC (CNCs-TDI) surface using HCl-acidified DMSO to form ammonium chloride groups. This step was also optimized by the authors and published elsewhere [49]. After carbamation and hydrolysis, the surface of the CNCs was fully covered with toluidine-like molecules (CNCs-TDN), which were then polymerized using the oxidizing agent, APS, forming an electrically conductive poly(toluidine)-like shell around the CNCs leading to novel electrically conductive CNCs (E-CNCs) with a core-shell nanostructure.

**Figure 4:**
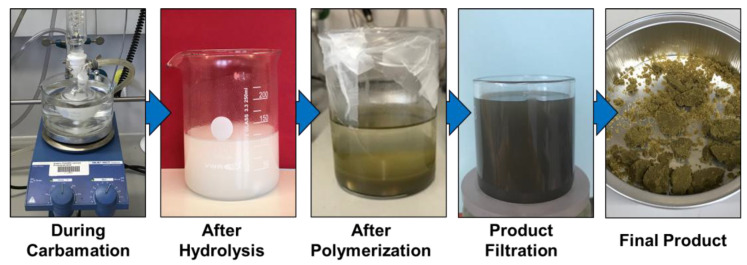
The change in CNCs’ color after carbamation, hydrolysis, polymerization, and filtration.

In more detail, the carbamation reaction between TDI and CNCs was optimized to allow the carbamation of all the reactive hydroxyl groups on the CNC surface with 2,4-TDI through its para isocyanate groups and keeping the ortho isocyanate groups unreacted. Toluene was used as a non-swelling solvent to allow the reaction to happen only on the CNC surface. Based on the work of Nishiyama on determining the crystallographic parameters of Cellulose I crystallites, it is possible to estimate the amount and percentage of the hydroxyl groups on the CNC surface based on its thickness [54]. The CNCs used in this work had a thickness of 7.0 ± 1.6 nm as measured using AFM. They are, therefore, made of ca. 14 × 14 chains of cellulose, 52 of which are supposed to be on the surface. These 52 chains have 168 hydroxyl groups on the surface compared to a total of 1176 per a cross-section unit (cellubiose unit). Therefore, the surface hydroxyl groups represent 14.3% (168/1176 * 100%) of the total hydroxyls of the CNCs. One third of this value represents the C3 hydroxyls groups (4.8%), which are unreactive due to inductive and steric hindrances and due to their involvement in hydrogen bonding. It is also known that a significant fraction of C6 hydroxyls are sulfonated by the action of sulfuric acid during the production of the CNCs. Using elemental analysis, it was estimated that 42% of C6 hydroxyls were sulfonated, which represent 2.0% of the total CNC surface hydroxyls. Therefore, only 7.5% (14.3 − 4.8 − 2.0 = 7.5) of the total CNC hydroxyls are reactive on the surface, representing the maximum possible degree of substitution (DS_max_). Using a carbamation temperature of 35 °C and a TDI/CNCs molar ratio of 3 (Figure 4), the degree of carbamation (DS_TDI_) was 7.7 ± 0.1% as estimated using elemental analysis, which is comparable to the DS_max_ (7.5%) estimated earlier. This suggests that all the reactive surface hydroxyls were carbamated. The next step was the hydrolysis of the ortho isocyanates of the grafted TDI to amine groups using HCl-acidified dimethylsulfoxide as a hydrolysis medium according to the method of Abushammala [49]. Following this method, it is possible to both hydrolyze the surface ortho isocyanates and quantify them based on the pH value obtained upon hydrolysis. The results showed that the degree of NCO substitution (DS_NCO_) was 7.2 ± 0.3% (1.33 ± 0.07 mmol of free isocyanates) resulting in a maximum DS_NCO_/DS_TDI_ of 93%, which means that 93% of the 2,4-TDI placed on the CNC surface had unreacted ortho isocyanates, which were then hydrolyzed to amine groups using the hydrolysis medium. After hydrolysis, APS was added to the resultant CNCs to initiate the polymerization of the toluidine-like (TDN) molecules that were generated on the CNCs-TDN surface upon carbamation and hydrolysis. Directly after the APS addition, the white CNCs-TDN started to change in color to yellow then to green in a similar behavior of aniline and toluidine polymerization to form poly(aniline) and poly(toluidine), respectively. After 48 h, no further change in color could be observed and the resultant green CNCs (E-CNCs) were collected by filtration, washed, and left to dry under vacuum (Figure 4). The E-CNCs were then characterized for their chemical, morphological, and most importantly for their electrical properties.

The elemental composition of the CNCs and the mass yield at each process stage were used to monitor the chemical changes on the CNC surface (Table 1). The original CNCs expectedly consisted mostly of carbon and oxygen atoms since hydrogen cannot be detected using EDX. Some sulfur and sodium were detected indicating some sodium sulfonate groups, which are a result of the reaction of sulfuric acid with the C6 hydroxyl group on the CNC surface during the preparation of the CNCs from pulp. After carbamation using 2,4-TDI, the CNCs-TDI had 4.2% of nitrogen and its carbon content increased as its oxygen content decreased, confirming the reaction of TDI molecules with CNCs, since TDI is richer in both carbon and nitrogen but poorer in oxygen compared to the original CNCs. The reaction of TDI with CNCs expectedly also diluted the amounts of S and Na. The nitrogen and carbon content of the CNCs-TDI could be used to estimate DS_TDI_, which was found to be 7.7%. In terms of reaction yield, 1.25 g of CNCs-TDI was collected, starting with 1.0 g of CNCs. Assuming no mass losses, this means that 1.0 g of CNCs (equivalent to 6.2 mmol of anhydroglucose units) reacted with 0.25 g of 2,4-TDI (equivalent to 1.4 mmol of 2,4-TDI). This results in an estimated DS_TDI_ of 7.9%, which is in agreement with the DS_TDI_ estimated using elemental analysis (7.7%). After the hydrolysis of CNCs-TDI using HCl-acidified DMSO, it was expected that the ortho-N=C=O groups of the surface TDI molecules will become -NH_3_Cl and CO_2_ gas will be released. This was evident from the elemental analysis results of CNCs-TDN as the carbon and oxygen contents decreased as the chloride content increased from 0 to 5.3% and no significant changes in N, S, and Na contents were observed. Mass yield increased slightly because of the chloride content, which has a higher atomic weight than carbon and oxygen combined. After the polymerization of the CNCs-TDN surface using APS, no major changes in the C, O, N, S, and Na contents were observed but the Cl content dropped significantly. Most probably it was a result of nitrogen partial deprotonation during the formation of the quinoids and benzenoids of the conductive shell. This led to a small drop in the mass yield to 1.27 g starting with 1.31 g.

**Figure 5:**
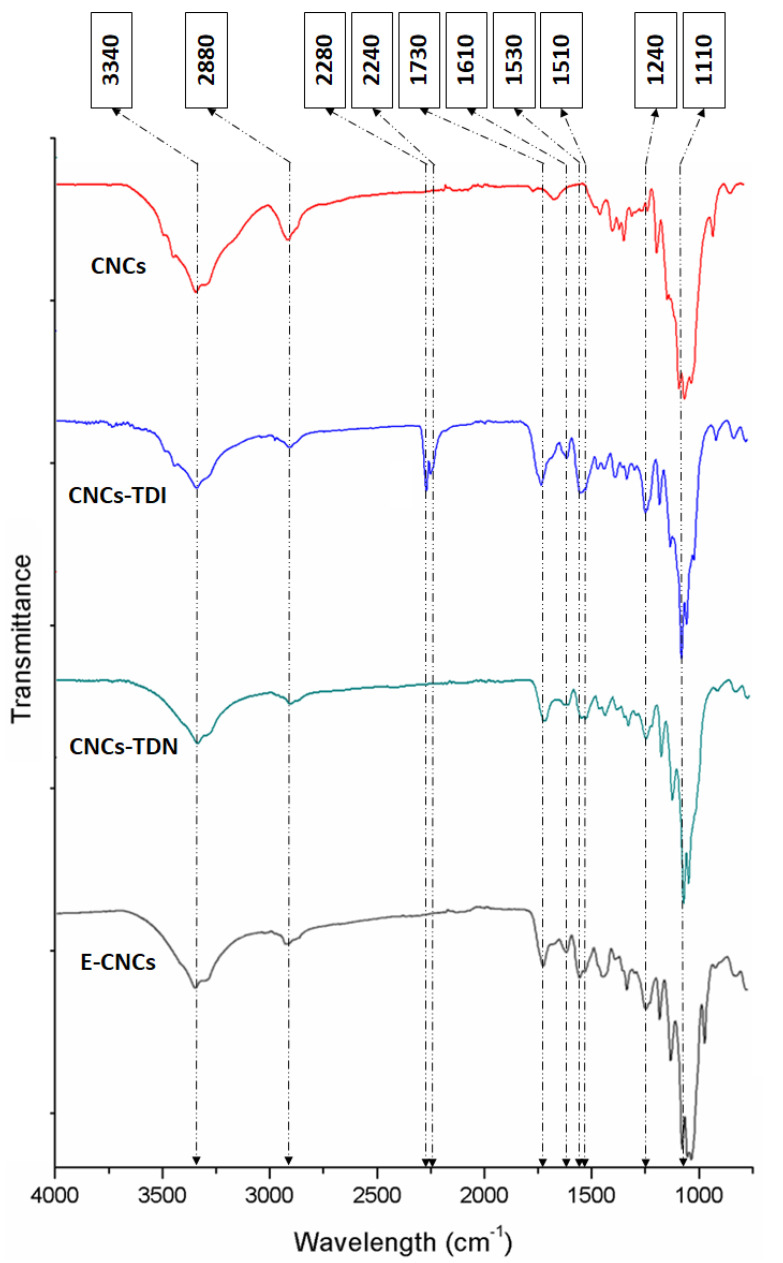
FT-IR spectra of the original CNCs, after carbamation using 2,4-TDI (CNCs-TDI), hydrolysis using HCl-acidified DMSO (CNCs-TDN), and after polymerization using APS (E-CNCs).

The elemental analysis and mass yield results provided a good insight on what could have happened during the carbamation, hydrolysis, and polymerization reactions on the CNC surface, but FT-IR can provide more details on what bonds were formed and what bonds were broken during these reactions (Figure 5). Carbamation can be confirmed by the appearance of the N=C=O asymmetric vibration bands around 2240 and 2280 cm^−1^, the urethane carbonyl stretching vibration band at 1730 cm^−1^, the urethane N-H stretching vibration band at 1530 cm^−1^, the urethane C-N stretching vibration band at 1240 cm^−1^, and the vibration bands of the aromatic ring at 1510 and 1610 cm^−1^ [55]. The original CNCs showed the typical glycosidic vibration bands ca. 1110 cm^−1^, the C-H stretching vibration band at 2880 cm^−1^, and the O-H stretching vibration band at 3340 cm^−1^. Upon carbamation, the vibration bands of the ortho-isocyanate, carbonyl and amide of the urethane bond, and the aromatic ring appeared, indicating that the 2,4-TDI molecules reacted with the CNCs through their para-NCO. After hydrolysis, the ortho-NCO vibration bands at 2240 and 2280 cm^−1^ expectedly disappeared as the NCO groups were hydrolyzed to amines. It was, however, not possible to detect the N-H stretching vibration band of the generated amine groups since it overlapped with the O-H stretching vibration band of the CNCs at 3340 cm^−1^. After polymerization, no significant changes were possible to detect using FT-IR because the main formed bonds, such as C-N stretching in benzenoid and quinoid rings, overlap with the C=C bands of the aromatic ring at 1510 and 1610 cm^−1^. It is important to remember that the shell around the CNCs in terms of mass is less dominant compared to the CNCs themselves as the CNC core has higher weight, which is evident in the mass yield data in Table 1.

**Figure 6:**
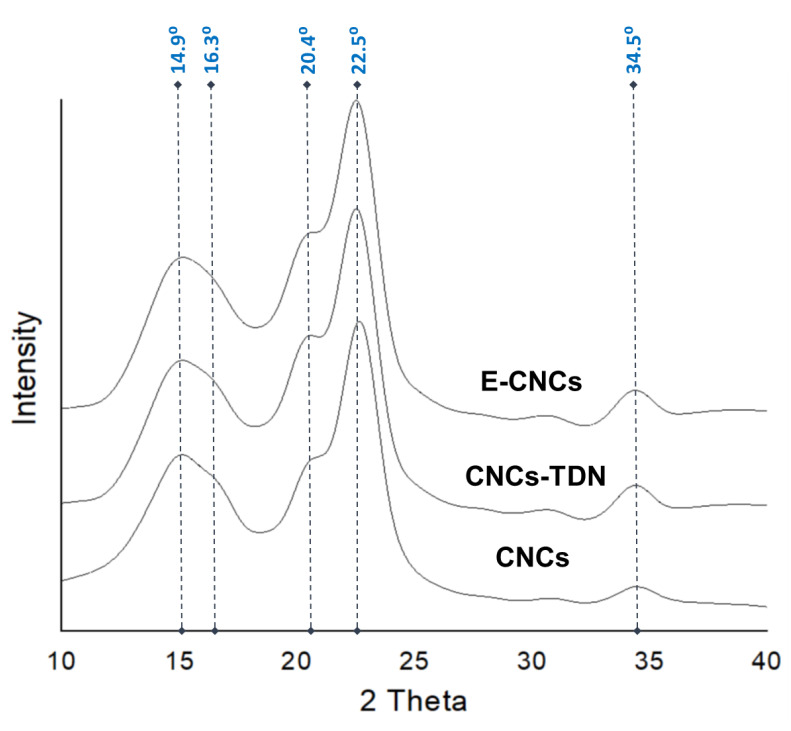
The crystalline structure of the original CNCs after hydrolysis using HCl-acidified DMSO (CNCs-TDN) and after polymerization using APS (E-CNCs).

To confirm that the carbamation, hydrolysis, and polymerization reactions all took place on the surface of CNCs and not inside them, the CNCs were characterized before and after these processes using X-ray diffraction. The results showed that the CNCs had the native crystalline structure of cellulose (Cellulose I), which exhibits five diffraction peaks at 2θ of 14.8°, 16.3°, 20.4°, 22.4°, and 34.5° corresponding to the diffractions of the crystalline planes 101, 10-1, 021, 200, and 040 [56] (Figure 6). No Cellulose II crystalline structure was detected at 2θ of 12° indicating that no cellulose dissolution and regeneration took place. Using peak deconvolution, the crystallinity of the CNCs was determined based on the areas of the five diffraction peaks and the amorphous peak at 2θ of 18°. The results showed that the crystallinity slightly dropped from 90.2% ± 1.3% for the original CNCs to 87.2% ± 1.4% for the CNCs-TDN to 86.6% ± 1.6% for the E-CNCs confirming that the carbamation, hydrolysis, and polymerization reactions all took place on the CNC surface. If any of these reactions took place inside the crystal, they would have led to a significant drop in crystallinity in addition to a possible formation of Cellulose II crystals.

**Figure 7:**
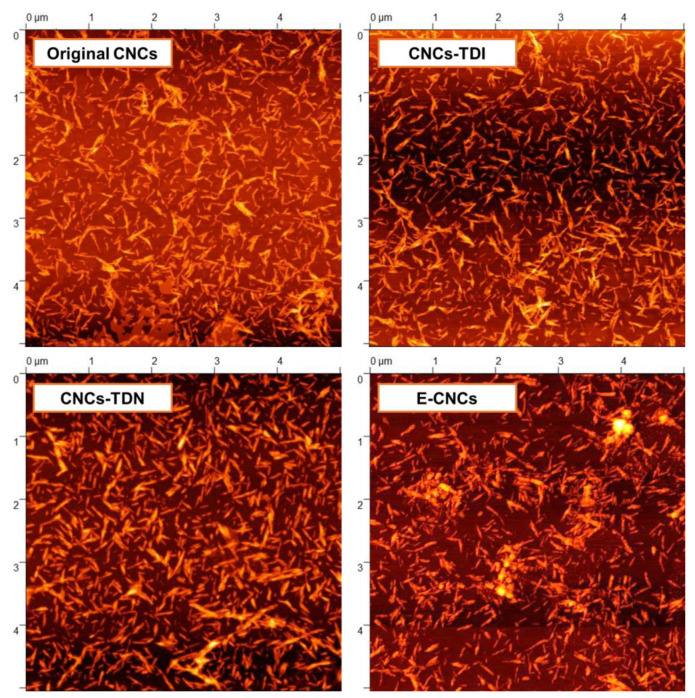
AFM topography images of the original CNCs, after carbamation using 2,4-TDI (CNCs-TDI), hydrolysis using HCl-acidified DMSO (CNCs-TDN), and after polymerization using APS (E-CNCs).

Atomic force microscopy (AFM) was then used to confirm the morphology of the CNCs after carbamation (CNCs-TDI), after hydrolysis (CNCs-TDN), and after polymerization (E-CNCs) (Figure 7). The AFM images showed that the CNCs maintained their typical nanorod morphology. The original CNCs had a thickness of 7.0 ± 1.6 nm, which increased to 7.8 ± 1.7 nm upon carbamation and to 8.5 ± 1.1 nm upon hydrolysis. The E-CNCs had a thickness of 8.7 ± 1.9 nm. This increase in thickness compared to the original CNCs could be a result of the poly(toluidine)-like shell introduced to the CNC surface. The AFM images also showed that the CNCs were well-dispersed at all stages with no observed agglomeration or stackings, indicating that no major crosslinking reactions took place between the CNC particles.

**Figure 8:**
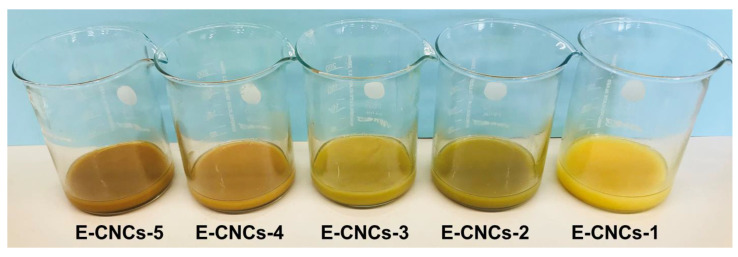
The colors of the E-CNCs after polymerization using different amounts of APS showing the different chromic forms of the conductive shell.

Finally, and most importantly, the electrical conductivity of the E-CNCs were measured using the four-point method as shown in Figure 2 and the impact of APS amount on the properties of the E-CNCs was investigated. The results showed that using an APS/TDN molar ratio of 1.25 (using 379 mg of APS) led to the highest electrical conductivity of 0.46 S/cm since this ratio was expected to lead to the formation of the emeraldine salt form of the polymerized TDN on the CNC shell, which is in agreement with the obtained green color (Table 2 and Figure 8). Using a lower amount of APS (304 mg, APS/TDN molar ratio of 1.00) led to the formation of the insulated yellow-colored leucomeraldine (E-CNCs-1) form whereas using higher amounts of 410 and more led to over-oxidation of the shell causing the brown color (E-CNCs-4 and E-CNCs-5). This brown color has been reported in the literature for over-oxidized poly(aniline) [57,58]. The conductivity values and colors are in agreement with the literature reports, which obtained the highest conductivity using an APS/TDN molar ratio of 1–1.25 [59,60].

## 4. Conclusions

Novel electrically conductive CNCs were prepared by subjecting neat CNCs to surface carbamation using 2,4-TDI followed by hydrolysis of the unreacted isocyanates of the 2,4-TDI molecules to amine groups using HCl-acidified DMSO. The resultant toluidine-like molecules on the CNC surface were then polymerized using APS leading to a green electrically conductive shell around each CNC as confirmed using elemental analysis, FT-IR, X-ray diffraction, AFM, and conductivity measurements. The FT-IR and elemental analysis results confirmed the expected chemical changes upon carbamation, hydrolysis, and polymerization and X-ray diffraction confirmed the permanence of the native crystalline structure of the CNCs despite a small drop in crystallinity. AFM showed that the electrically conductive CNCs were on average slightly thicker than the original ones, possibly due to the growth of the conductive shell around them. The obtained CNCs had a maximum electrical conductivity of 0.46 S/cm, which was strongly dependent on the amount of APS used for surface polymerization. Such electrically conductive CNCs open several doors for the utilization of CNCs in a wider range of applications including biodegradable electronics and biosensing. Such CNCs have never been reported in the literature and are currently being patented as indicated in the section below.

## 5. Patents

A patent has resulted from this research work entitled “Process for preparing individual cellulose nanocrystals, and cellulose nanocrystals and use thereof” under the following applications: US20220135775A, EP3924416A, DE102019103717A, CA3126442A, JP2022521145A [61].

## Figures and Tables

**Table 1:** The elemental composition of the CNCs and mass yield (starting with 1.0 g of CNCs) after carbamation using 2,4-TDI (CNCs-TDI), hydrolysis using HCl-acidified DMSO (CNCs-TDN), and polymerization using APS (E-CNCs).

Sample	Elemental Composition (%)						Mass Yield (g)
	%C	%O	%N	%S	%Na	%Cl	
Original CNCs	45.9%	52.1%	0.0%	1.2%	0.8%	0.0%	1.0
CNCs-TDI	54.1%	40.1%	4.2%	0.8%	0.6%	0.0%	1.25
CNCs-TDN	51.3%	38.1%	4.0%	0.7%	0.7%	5.3%	1.31
E-CNCs	52.3%	39.2%	4.1%	0.8%	0.6%	3.0%	1.27

**Table 2:** The color and electrical conductivity of the E-CNCs after polymerization using different amounts of APS.

Sample	mmol TDN (mmol)	APS/TDN Molar Ratio	Used Mass of APS (mg)	Color	Electrical Conductivity (S/cm)
E-CNCs-1	1.33 ± 0.07	1.00	304	Yellow	1.0 × 10^−4^
E-CNCs-2	1.33 ± 0.07	1.25	379	Dark Green	0.46
E-CNCs-3	1.33 ± 0.07	1.35	410	Green/Brown	4.9 × 10^−3^
E-CNCs-4	1.33 ± 0.07	1.50	455	Light Brown	7.3 × 10^−5^
E-CNCs-5	1.33 ± 0.07	1.75	531	Brown	1.8 × 10^−5^

## Data Availability

Not applicable.
